# Systemic Inflammation and Oxidative Stress Markers in Patients With Medication Overuse Headache

**DOI:** 10.1002/brb3.71004

**Published:** 2025-10-20

**Authors:** Changling Li, Peiqi He, Yanbo Li, Mengmeng Ma, Ning Chen, Qian Liu, Yang Zhang, Xin Jiang, Shiqin Li, Hui Lang, Jinghuan Fang, Li He

**Affiliations:** ^1^ Department of Neurology West China Hospital of Sichuan University Chengdu Sichuan China

**Keywords:** inflammation, medication overuse headache, oxidative stress, peripheral markers

## Abstract

**Background:**

Recent scientific evidence has suggested that inflammation and oxidative stress may play a significant role in the pathogenesis of medication overuse headache (MOH). This study aims to investigate the differences in peripheral markers of systemic inflammation and oxidative stress, such as the monocyte to high‐density lipoprotein (HDL) ratio (MHR), and their correlation with clinical symptoms in MOH patients, episodic migraine patients, and healthy controls.

**Methods:**

This cross‐sectional study recruited MOH patients along with age‐ and sex‐matched controls. Differences in indicators of inflammation and oxidative stress were compared across different groups, including MOH patients with different preexisting headache subtypes. Univariate and multivariate logistic regression analyses were employed to identify independent factors associated with the occurrence of MOH.

**Results:**

The study comprised 109 MOH patients, 68 episodic migraine patients, and 86 healthy controls. MOH patients exhibited significantly higher values of MHR, lymphocyte to HDL ratio (LHR), and neutrophil to HDL ratio (NHR) compared to healthy controls. No statistically significant differences in these indicators were observed between the MOH subgroups. Correlation analysis revealed that only the triglyceride‐glucose index was negatively correlated with monthly headache days in MOH patients (*r* = ‐0.230, *p* = 0.016). Additionally, elevated values of MHR odds ratios ((OR) = 2.32, 95% confidence intervals (CI) 1.18–4.56), LHR (OR = 2.56, 95% CI 1.40–4.67), and NHR (OR = 2.09, 95% CI 1.16–3.75) were independently associated with MOH.

**Conclusion:**

MOH patients demonstrated markedly distinct inflammatory and oxidative stress profiles. Elevated values of LHR, MHR, and NHR were independently associated with MOH.

## Graphical abstract

Patients with medication overuse headache (MOH) showed distinct inflammatory and oxidative stress profiles compared to healthy controls. Elevated values of lymphocyte to high‐density lipoprotein (HDL) ratio (LHR), monocyte to HDL ratio (MHR), and neutrophil to HDL ratio (NHR) were independently associated with MOH.

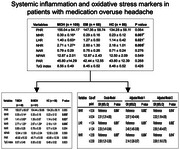

**Abbreviations**
ANOVA: One‐way analysis of variance
AUC: area under the ROC curve
CI: 95% confidence intervals
EM: episodic migraine
FPG: fasting plasma glucose
HC: healthy controls
HDL: high density lipoprotein
HDL‐C: high density lipoprotein cholesterol
ICHD‐III: International Classification of Headache Disorders, third edition
LDL‐C: low‐density lipoprotein cholesterol
LHR: lymphocyte to high‐density lipoprotein ratio
MHR: monocyte to high‐density lipoprotein ratio
M‐MOH: medication overuse headache with a preexisting diagnosis of migraine
MOH: medication overuse headache
NAR: neutrophil to albumin ratio
NHR: neutrophil to high‐density lipoprotein ratio
NPAR: neutrophil percentage‐to‐albumin ratio
NSAIDs: nonsteroidal anti‐inflammatory drugs
OR: odds ratios
PAR: platelet to albumin ratio
PHR: platelet to high‐density lipoprotein ratio
ROC: receiver operating characteristic curve
TC: total cholesterol
TG: triglyceride
T‐MOH: medication overuse headache with a preexisting diagnosis of tension‐type headache
TTH: tension‐type headache
TyG: triglyceride‐glucose

## 1 Introduction

Medication overuse headache (MOH) is a chronic secondary headache disorder resulting from the excessive use of acute headache medications, such as nonsteroidal anti‐inflammatory drugs (NSAIDs), triptans, and combination products, by individuals with a preexisting headache condition (Ashina et al. 2023; Diener et al. 2019). MOH is consistently linked to significant disability and reduced quality of life, imposing a substantial burden on both individuals and society (Green 2021; Vandenbussche et al. 2018). Despite this, the pathophysiological mechanisms underlying the development of MOH remain a topic of ongoing debate. Investigating biomarkers for MOH holds significant clinical potential and may enhance our understanding of the disorder's underlying mechanisms.

Recent scientific evidence indicates that inflammation and oxidative stress may play a crucial role in the pathogenesis of MOH (De Luca et al. 2021; Lucchesi et al. 2015; Vuralli et al. 2024; Wang et al. 2023). On one hand, the release of vasoactive pro‐inflammatory factors appeared to be a primary driver of neuroinflammation (Dalkara et al. 2010). MOH patients exhibited a low‐grade systemic inflammatory state, evidenced by a significant increase in serum levels of pro‐inflammatory cytokines, including IL‐6, IL‐1β, and others (Sun‐Edelstein et al. 2021; Vuralli et al. 2024; Vuralli, Ceren Akgor, Gok Dagidir, et al. 2024). On the other hand, oxidative stress, caused by an imbalance between antioxidant defense mechanisms and reactive oxygen/nitrogen species, results in damage to proteins, DNA, and lipids (Ardizzone et al. 2024; Karabulut et al. 2016). Among these effects, epigenetic DNA methylation alterations were associated with headache chronification and MOH (Carlsen et al. 2023; Winsvold et al. 2018). MOH patients also displayed changes in markers of oxidative stress, including increased levels of advanced oxidation protein products and decreased levels of thiol groups and ferric reducing antioxidant power (Dini et al. 2019; Lucchesi et al. 2015). Treatment with onabotulinumtoxinA or targeted antibodies against the calcitonin gene‐related peptide and its receptors over a six‐month period was shown to improve plasma levels of oxidative stress biomarkers (De Luca et al. 2021; Dini et al. 2019).

Recent research has focused on elucidating the mechanisms underlying MOH from the perspectives of oxidative stress and inflammation, with blood biomarkers being the most commonly utilized methods for investigation. However, many biomarkers are costly to measure or are difficult to collect routinely. Consequently, there is increasing interest in identifying simple, cost‐effective, and readily accessible biomarkers that reflect systemic inflammation and oxidative stress. Combined markers derived from routine peripheral blood cell counts and biochemical tests, such as platelet to high‐density lipoprotein (HDL) ratio (PHR), monocyte to HDL ratio (MHR), lymphocyte to HDL ratio (LHR), neutrophil to HDL ratio (NHR), platelet to albumin ratio (PAR), neutrophil to albumin ratio (NAR), and the triglyceride‐glucose (TyG) index, have been proposed as potential indicators of systemic inflammation and oxidative stress in various inflammatory diseases (Gounden et al. 2024; Wei et al. 2024). These markers have shown significant associations with the presence and prognosis of several neurological conditions, including Parkinson's disease and ischemic stroke (Gkantzios et al. 2023; Z. Liu et al. 2021; Wei et al. 2024). Despite this, research on these combined markers in the context of migraine is limited (Y. Liu et al. 2023; Ulusoy 2020). Notably, alterations in these inflammatory parameters and their correlation with clinical symptoms have not been previously assessed in MOH patients.

Therefore, this study aims to investigate the differences in these indicators of systemic inflammation and oxidative stress among MOH patients, episodic migraine (EM) patients, and healthy controls (HC). Additionally, it seeks to explore the potential correlations between these indicators and the clinical symptoms of MOH (such as headache days per month, days per month with acute medication, etc.).

## 2 Methods

### 2.1 Participants

This cross‐sectional study included 109 MOH patients, 68 EM patients, and 86 HC. Between January 2021 and March 2024, headache specialists consecutively recruited MOH patients aged 18 to 65 years from a tertiary headache clinic at West China Hospital. The inclusion criteria for MOH patients were as follows: (1) a diagnosis of MOH according to the International Classification of Headache Disorders, third edition (ICHD‐III) criteria (IHS 2018); (2) a pre‐existing diagnosis of tension‐type headache (TTH) and migraine; and (3) no use of prophylactic medications in the preceding three months. Control groups consisted of the EM group and the HC group, which were matched for age and sex with the MOH patients. The following were the inclusion criteria for EM patients: (1) age between 18 and 65 years; (2) diagnosis according to the ICHD‐III criteria for migraine without aura (IHS 2018), with headache episodes occurring on 1–14 days per month; (3) stable episodic migraine status over the past three months; and (4) no use of prophylactic medications in the previous three months. HC were enrolled as healthy individuals aged 18 to 65 years with no history of headaches, confirmed by medical records and physical examination showing no significant abnormalities. Exclusion criteria for participants across MOH, EM, and HC groups were as follows: (1) Individuals lacking complete routine blood tests or biochemical profiles were excluded; and (2) individuals with other chronic pain disorders, acute or recent infections, major neuropsychiatric conditions, or systemic diseases were excluded.

### 2.2 Clinical Data Collection

Clinical data were obtained from participants during their initial visit to the clinic. The demographic information included age, sex, ethnicity, and the community of residence. For patients presenting with headaches, a structured questionnaire was administered to gather data regarding headache occurrences in the three months preceding their initial visit. This questionnaire encompassed headache characteristics, including headache history, location, intensity (assessed via the Visual Analogue Scale, ranging from 0 to 10), and duration. It also recorded the type and frequency of acute medication usage, monthly headache days, accompanying symptoms, and comorbidities.

Blood samples were collected by nurses at the outpatient clinic of West China Hospital after patients had fasted for 8–10 h. The blood indices measured included counts of white blood cells, monocytes, lymphocytes, neutrophils, platelets, and erythrocytes. The measurements also included levels of albumin, fasting plasma glucose (FPG), triglycerides (TG), high‐density lipoprotein cholesterol (HDL‐C), total cholesterol (TC), low‐density lipoprotein cholesterol (LDL‐C), and others. Blood cell counts and biochemical parameters were analyzed using an automated analyzer.

Systemic inflammation and oxidative stress indicators were explored as biomarkers to detect MOH or characterize its headache symptoms. These indicators were obtained from routine peripheral blood cell counts and biochemical tests, calculated using specific formulas (Lan et al. 2023; Song et al. 2023; Wei et al. 2024): (1) PHR = platelet count (10^9^/L)/HDL (mmol/L); (2) MHR = monocyte count (10^9^/L)/HDL (mmol/L); (3) LHR = lymphocyte count (10^9^/L)/HDL (mmol/L); (4) NHR = neutrophil count (10^9^/L)/HDL (mmol/L); (5) NAR = neutrophil count (10^9^/L)/albumin (g/dL); (6) NPAR = neutrophil percentage (%) × 100 / albumin (g/dL); (7) PAR = platelet count (10^9^/L)/albumin (g/dL); and (8) TyG index = Ln [fasting TG (mg/dL) × fasting plasma glucose (mg/dL)/2]. These seven ratios (NHR, LHR, MHR, PHR, NAR, PAR, and NPAR) serve as composite markers reflecting systemic inflammation and oxidative stress by balancing pro‐inflammatory cell counts (neutrophils, lymphocytes, monocytes, and platelets) against anti‐inflammatory/antioxidant markers (HDL and albumin), while the TyG index is a marker of insulin resistance, which promotes oxidative stress and inflammation (Gounden et al. 2024; Lan et al. 2023; Wei et al. 2024). Changes in these indicator levels might be useful for identifying MOH.

### 2.3 Statistical Analyses

Data analysis was conducted using SPSS Software, version 22.0. The Shapiro–Wilk test assessed the normality of quantitative variables. Continuous variables were reported as means ± standard deviation. When analyzing normally distributed continuous variables, one‐way analysis of variance (ANOVA) was utilized for three groups, while a student *t*‐test was used for two groups. When analyzing non‐normally distributed continuous variables, the Kruskal–Wallis test was employed for three groups, while the Mann–Whitney *U* test was performed for two groups. Following the Kruskal–Wallis test and ANOVA, the Bonferroni multiple comparisons test was applied for post hoc analysis. Categorical variables were presented as counts (percentages), with group differences assessed using chi‐square or Fisher's exact test. Spearman rank correlation analyzed correlations between inflammatory indicators and clinical characteristics in MOH patients. The receiver operator characteristic (ROC) curve was utilized to determine the cut‐off values for the MHR, LHR, and NHR by maximizing the Youden index (sensitivity + specificity − 1), and to assess their diagnostic efficacy for MOH. Univariate and multivariate logistic regression analyses identified predictors of MOH, calculating odds ratios (OR) and 95% confidence intervals (CI).

A *p*‐value < 0.05 was deemed statistically significant, and all tests were two‐tailed.

## 3 Results

### 3.1 Comparison Among MOH, EM, and HC

**FIGURE 1:**
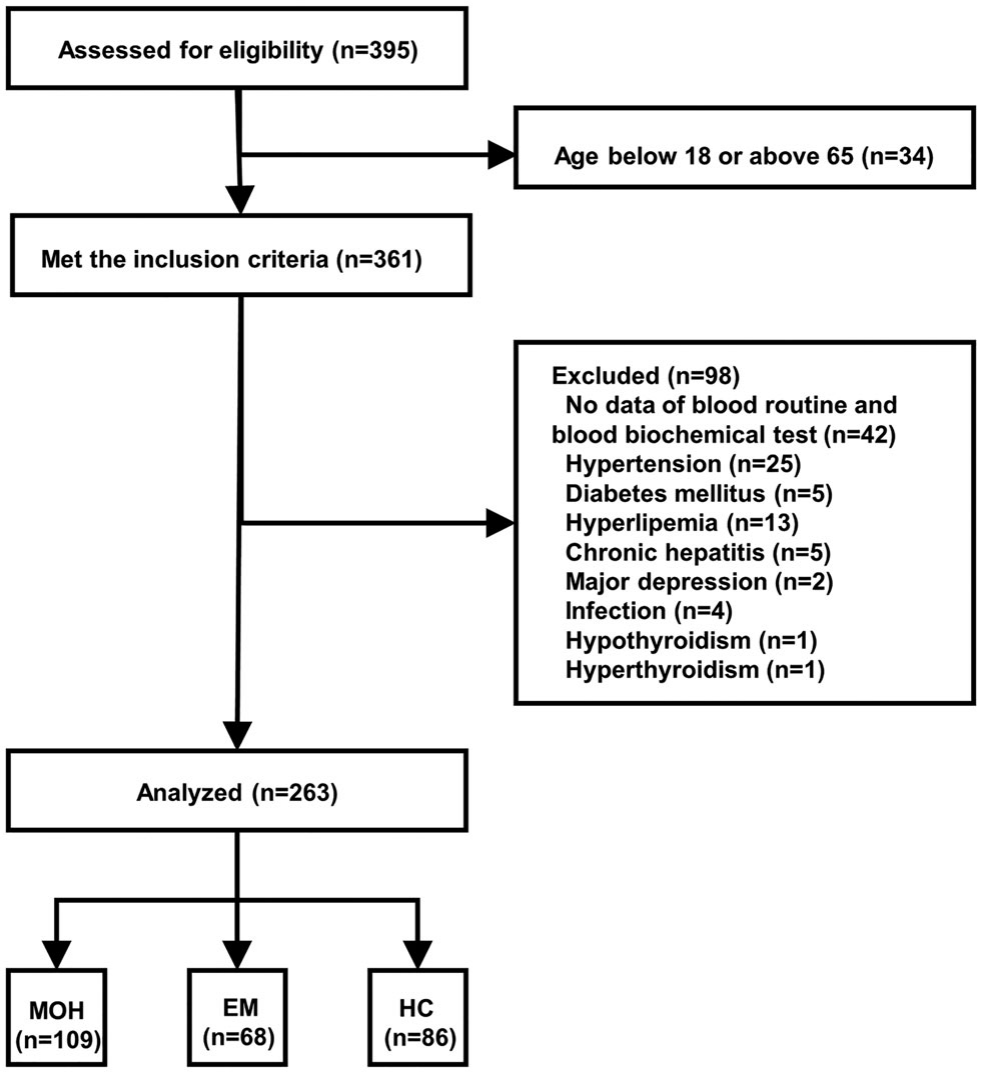
Flowchart detailing the study population inclusion process. Abbreviations: EM, episodic migraine; HC, healthy controls; MOH, medication overuse headache.

The flowchart presented in Figure 1 illustrates the inclusion process for the study population. Finally, the study encompassed a total of 109 MOH patients (79 females; mean age 48.94 ± 8.42 years), 68 EM patients (46 females; mean age 46.57 ± 8.44 years), and 86 HC (57 females; mean age 48.62 ± 6.58 years). The groups were well‐matched in terms of age (*p* = 0.177) and sex (*p* = 0.616), as detailed in Table 1. Among the MOH patients, 61.5% had preexisting headache diagnoses of episodic migraine and TTH. At the onset of headache, 97 patients (89.0%) opted to use combination analgesics, primarily comprising aspirin, acetaminophen, caffeine, aminopyrine, and phenacetin. Compared to EM patients, MOH patients exhibited a significantly longer duration of headache (16.63 ± 10.29 vs. 12.75 ± 6.95, *p* = 0.016), more headache days per month (23.97 ± 6.71 vs. 5.00 ± 2.72, *p* < 0.001), and increased days per month with acute medication (21.44 ± 7.62 vs. 2.84 ± 1.40, *p* < 0.001).

**TABLE 1:** Comparison of clinical features and inflammatory indicators among MOH, EM, and HC groups.

Variables	MOH (*n* = 109)	EM (*n* = 68)	HC (*n* = 86)	*p*‐value
Age, year	48.94 ± 8.42	46.57 ± 8.44	48.62 ± 6.58	0.177
Sex, female	79 (72.5%)	46 (67.6%)	57 (66.3%)	0.616
Duration of headache, year	16.63 ± 10.29	12.75 ± 6.95	——	0.016^*^
Headache days per month	23.97 ± 6.71	5.00 ± 2.72	——	< 0.001^*^
Headache intensity per month	6.07 ± 1.25	6.32 ± 1.13	——	0.161
Days per month with acute medication	21.44 ± 7.62	2.84 ± 1.40	——	< 0.001^*^
Preexisting headache diagnoses				
Chronic migraine	7 (6.4%)	——	——	
Episodic migraine and TTH	67 (61.5%)	——	——	
Chronic TTH	35 (32.1%)	——	——	
Duration of medication overuse, year	4.53 ± 5.23	——	——	
Type of medication overuse				
Simple analgesics	10 (9.2%)	——	——	
Triptans	2 (1.8%)	——		
Compound analgesics	97 (89.0%)	——	——	
Platelet, 10^9^/L	210.67 ± 64.57	197.22 ± 59.24	203.42 ± 57.85	0.352
White blood cells				
Monocytes, 10^9^/L	0.40 ± 0.17	0.38 ± 0.15	0.36 ± 0.13	0.230
Lymphocytes, 10^9^/L	1.86 ± 0.57	1.70 ± 0.43	1.76 ± 0.48	0.146
Neutrophils, 10^9^/L	3.65 ± 1.38	3.47 ± 1.20	3.31 ± 1.10	0.358
ALB, g/L	46.18 ± 2.88	46.52 ± 3.00	46.46 ± 2.75	0.541
FPG, mmol/L	4.98 ± 0.64	4.96 ± 0.48	5.25 ± 0.85	0.123
TG, mmol/L	1.50 ± 0.93	1.36 ± 0.78	1.29 ± 0.60	0.259
HDL‐C, mmol/L	1.44 ± 0.38^a^	1.43 ± 0.34	1.73 ± 0.91	0.037^*^
TC, mmol/L	4.85 ± 0.88	4.77 ± 0.90	4.94 ± 0.81	0.480
LDL‐C, mmol/L	2.89 ± 0.78	2.86 ± 0.78	3.02 ± 0.69	0.194
PHR	156.04 ± 64.17	147.36 ± 58.74	134.28 ± 58.11	0.064
MHR	0.30 ± 0.16^a^	0.29 ± 0.16	0.23 ± 0.12	0.007^*^
LHR	1.40 ± 0.63^a^	1.27 ± 0.50	1.14 ± 0.42	0.021^*^
NHR	2.71 ± 1.27^a^	2.60 ± 1.30	2.18 ± 1.01	0.005^*^
NAR	0.79 ± 0.29	0.75 ± 0.26	0.71 ± 0.24	0.276
NPAR	12.87 ± 2.01	12.87 ± 2.43	12.50 ± 2.09	0.549
PAR	45.80 ±14.29	42.44 ± 12.55	43.80 ± 12.39	0.268
TyG index	8.56 ± 0.49	8.45 ± 0.52	8.48 ± 0.52	0.426

*Note*: Values are presented as absolute numbers (percentages), or mean ± standard deviation.

Abbreviations: ALB, albumin; EM, episodic migraine; FPG, fasting plasma glucose; HC, healthy controls; HDL‐C, high density lipoprotein cholesterol; LHR, lymphocyte to high‐density lipoprotein ratio; LDL‐C, low‐density lipoprotein cholesterol; MHR, monocyte to high‐density lipoprotein ratio; MOH, medication overuse headache; NAR, neutrophil to albumin ratio; NHR, neutrophil to high‐density lipoprotein ratio; NPAR, neutrophil percentage‐to‐albumin ratio; PAR, platelet to albumin ratio; PHR, platelet to high‐density lipoprotein ratio; TC, total cholesterol; TG, triglyceride; TTH, tension‐type headache; TyG, triglyceride‐glucose. ^*^Statistically significant at *p* < 0.05; ^a^vs. HC group, adjusted *p* < 0.05.

The analysis revealed statistically significant differences in the levels of HDL‐C, MHR, LHR, and NHR among the MOH, EM, and HC groups (p < 0.05). In post hoc pairwise comparisons, MOH patients demonstrated significantly elevated MHR (0.30 ± 0.16 vs. 0.23 ± 0.12, adjusted p = 0.007), LHR (1.40 ± 0.63 vs. 1.14 ± 0.42, adjusted p = 0.016), and NHR (2.71 ± 1.27 vs. 2.18 ± 1.01, adjusted *p* = 0.005), along with reduced HDL‐C levels (1.44 ± 0.38 vs. 1.73 ± 0.91, adjusted p = 0.043) compared to HC. No significant differences were observed in platelet count, white blood cell count, albumin, TG, TC, FPG, LDL‐C, PHR, NAR, NPAR, PAR, and TyG index across the three groups, as shown in Table 1.

### 3.2 Comparison Among T‐MOH, M‐MOH and HC

In this study, MOH patients were categorized into subgroups based on a preexisting diagnosis of TTH (T‐MOH) or migraine (M‐MOH). No significant differences in age or sex were observed among the T‐MOH, M‐MOH, and HC groups. The T‐MOH subgroup exhibited significantly more headache days per month (25.67 ± 6.14 vs. 22.64 ± 6.88, *p* = 0.017) and lower headache intensity per month (5.48 ± 1.30 vs. 6.54 ± 0.99, *p* < 0.001) compared to the M‐MOH subgroup, while no significant differences were observed in other clinical characteristics between these two subgroups (Table 2).

**TABLE 2:** Comparison of clinical features and inflammatory indicators among T‐MOH, M‐MOH, and HC groups.

Variables	T‐MOH (*n* = 48)	M‐MOH (*n* = 61)	HC (*n* = 86)	*p*‐value
Age, year	48.96 ± 7.13	48.92 ± 9.38	48.62 ± 6.58	0.778
Sex, female	34 (70.8%)	45 (73.8%)	57 (66.3%)	0.611
Duration of headache, year	15.53 ± 10.30	17.49 ± 10.29	——	0.244
Headache days per month	25.67 ± 6.14	22.64 ± 6.88	——	0.017^*^
Headache intensity per month	5.48 ± 1.30	6.54 ± 0.99	——	< 0.001^*^
Days per month with acute medication	21.17 ± 8.20	21.66 ± 7.20	——	0.697
Duration of medication overuse, year	4.62 ± 6.39	4.47 ± 4.15	——	0.171
Type of medication overuse				0.666
Simple analgesics	4 (8.3%)	6 (9.8%)	——	
Triptans	0 (0.0%)	2 (3.3%)	——	
Compound analgesics	44 (91.7%)	53 (86.9%)	——	
Platelet, 10^9^/L	209.73 ± 68.23	211.41 ± 62.10	203.42 ± 57.85	0.712
White blood cells				
Monocytes, 10^9^/L	0.40 ± 0.15	0.39 ± 0.19	0.36 ± 0.13	0.158
Lymphocytes, 10^9^/L	1.87 ± 0.57	1.85 ± 0.56	1.76 ± 0.48	0.442
Neutrophils, 10^9^/L	3.69 ± 1.37	3.61 ± 1.40	3.31 ± 1.10	0.319
ALB, g/L	45.92 ± 3.02	46.38 ± 2.78	46.46 ± 2.75	0.543
FPG, mmol/L	4.98 ± 0.60	4.97 ± 0.67	5.25 ± 0.85	0.135
TG, mmol/L	1.47 ± 0.73	1.53 ± 1.07	1.29 ± 0.60	0.275
HDL‐C, mmol/L	1.42 ± 0.35	1.46 ± 0.41	1.73 ± 0.91	0.047^*^
TC, mmol/L	4.91 ± 0.79	4.79 ± 0.95	4.94 ± 0.81	0.580
LDL‐C, mmol/L	2.97 ± 0.70	2.83 ± 0.85	3.02 ± 0.69	0.281
PHR	158.07 ± 69.87	154.44 ± 59.86	134.28 ± 58.11	0.065
MHR	0.30 ± 0.14^a^	0.30 ± 0.18	0.23 ± 0.12	0.008^*^
LHR	1.41 ± 0.60^a^	1.39 ± 0.67	1.14 ± 0.42	0.021^*^
NHR	2.77 ± 1.25^a^	2.67 ± 1.29^a^	2.18 ± 1.01	0.006^*^
NAR	0.80 ± 0.28	0.78 ± 0.30	0.71 ± 0.24	0.204
NPAR	12.94 ± 1.99	12.81 ± 2.04	12.50 ± 2.09	0.589
PAR	45.92 ±15.32	45.71 ±13.55	43.80 ± 12.39	0.590
TyG index	8.57 ± 0.46	8.55 ± 0.52	8.48 ± 0.52	0.603

*Note*: Values are presented as absolute numbers (percentages), or mean ± standard deviation.

Abbreviations: ALB, albumin; FPG, fasting plasma glucose; HC, healthy controls; HDL‐C, high density lipoprotein cholesterol; LHR, lymphocyte to high‐density lipoprotein ratio; LDL‐C, low‐density lipoprotein cholesterol; MHR, monocyte to high‐density lipoprotein ratio; M‐MOH, medication overuse headache with a preexisting diagnosis of migraine; NAR, neutrophil to albumin ratio; NHR, neutrophil to high‐density lipoprotein ratio; NPAR, neutrophil percentage‐to‐albumin ratio; PAR, platelet to albumin ratio; PHR, platelet to high‐density lipoprotein ratio; TC, total cholesterol; TG, triglyceride; T‐MOH, medication overuse headache with a preexisting diagnosis of tension‐type headache; TyG, triglyceride‐glucose. ^*^Statistically significant at *p* < 0.05; ^a^vs. HC group, adjusted *p* < 0.05.

Statistically significant differences were identified in the levels of HDL‐C, MHR, LHR, and NHR among the T‐MOH, M‐MOH, and HC groups (*p* < 0.05). Further analysis revealed that in comparison to the HC group, the T‐MOH group demonstrated significantly elevated MHR (0.30 ± 0.14 vs. 0.23 ± 0.12, adjusted *p* = 0.010), LHR (1.41 ± 0.60 vs. 1.14 ± 0.42, adjusted *p* = 0.039), and NHR (2.77 ± 1.25 vs. 2.18 ± 1.01, adjusted p = 0.016), while the M‐MOH group showed a significantly higher NHR (2.67 ± 1.29 vs. 2.18 ± 1.01, adjusted *p* = 0.037). However, HDL‐C levels did not exhibit significant differences in the post hoc analysis following Bonferroni correction. No statistically significant differences in these parameters were detected between the T‐MOH and M‐MOH groups.

### 3.3 Correlation Analysis Between Inflammatory Indicators and Clinical Features

The TyG index was the only parameter found to be significantly negatively correlated with headache days per month (*r* = −0.230, *p* = 0.016) in MOH patients. No significant correleations were found between PHR, MHR, LHR, NHR, NAR, NPAR, PAR, and the clinical features of MOH.

### 3.4 Univariate and Multivariate Logistic Regression Models

**FIGURE 2:**
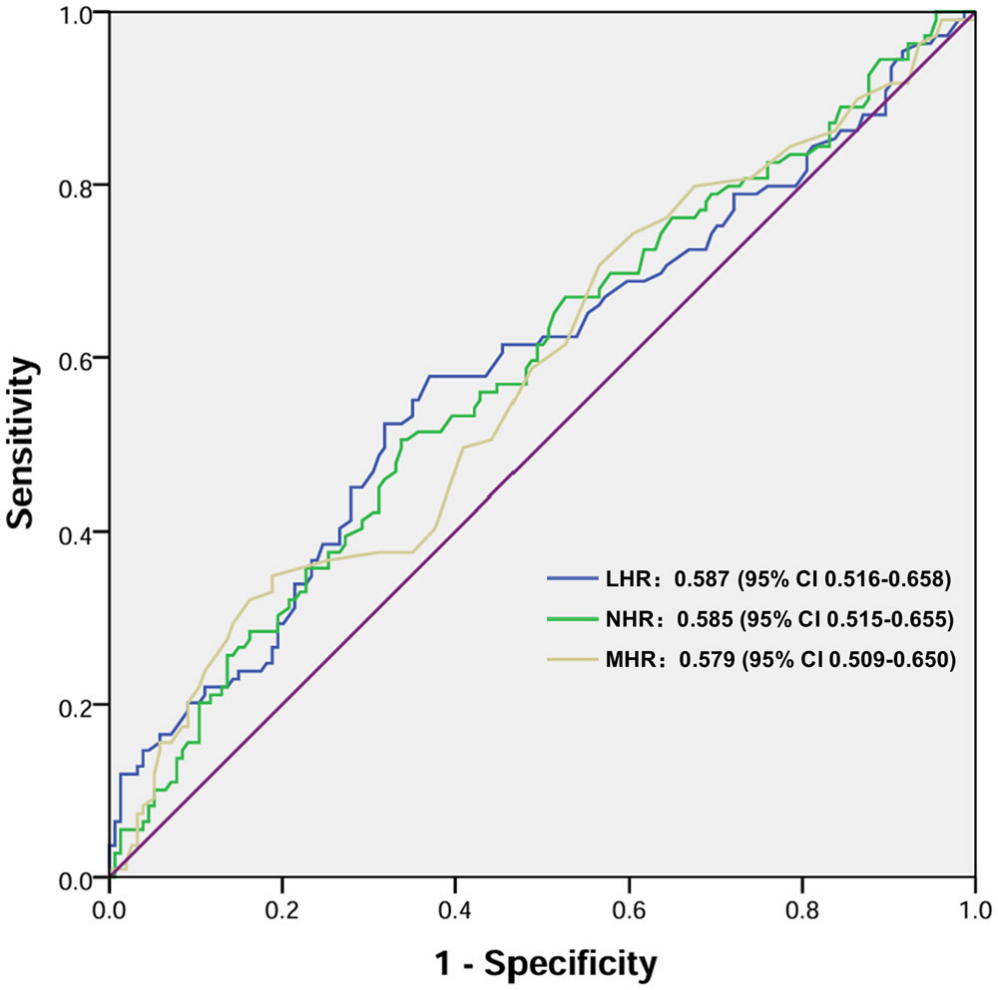
Comparison of the diagnostic efficacy of LHR, NHR, and MHR for MOH. Abbreviations: LHR, lymphocyte to high‐density lipoprotein ratio; MHR, monocyte to high‐density lipoprotein ratio; MOH, medication overuse headache; NHR, neutrophil to high‐density lipoprotein ratio.

The diagnostic efficacy of the LHR, NHR, and MHR in the context of MOH is illustrated in Figure 2. The area under the curve (AUC) for MHR in diagnosing MOH was determined to be 0.579 (95% CI 0.509–0.650, *p* = 0.029), with a cut‐off value of 0.34. The LHR demonstrated an AUC of 0.587 (95% CI 0.516–0.658, *p* = 0.016) for the diagnosis of MOH, accompanied by a cut‐off value of 1.24, while the NHR exhibited an AUC of 0.585 (95% CI 0.515–0.655, *p* = 0.036) with a cut‐off value of 2.58.

In univariate logistic regression analyses, elevated values of MHR (≥ 0.34), LHR (≥ 1.24), and NHR (≥ 2.58) were significantly associated with an increased risk of MOH (*p* < 0.05, Table 3). After controlling for age and sex, high values of MHR, LHR, and NHR remained associated with the risk of MOH (*p* < 0.05). Furthermore, following adjustment for age, sex, platelet count, albumin levels, TG, TC, FPG, and LDL‐C, elevated MHR (OR = 2.32, 95% CI 1.18–4.56, *p* = 0.014), LHR (OR = 2.56, 95% CI 1.40–4.67, *p* = 0.002), and NHR (OR = 2.09, 95% CI 1.16–3.75, *p* = 0.014) were independently associated with MOH.

**TABLE 3:** Logistic regression examining the association of MHR, LHR, and NHR with the risk of MOH.

Variables	Cut‐off point	Crude model		Adjusted model 1		Adjusted model 2	
		OR (95% CI)	*p*‐value	OR (95%CI)	*p*‐value	OR (95% CI)	*p*‐value
MHR	< 0.34	Reference	0.004^*^	Reference	0.002^*^	Reference	0.014^*^
	≥ 0.34	2.31 (1.31–4.06)		2.57 (1.42–4.64)		2.32 (1.18–4.56)	
LHR	< 1.24	Reference	0.001^*^	Reference	0.001^*^	Reference	0.002^*^
	≥ 1.24	2.33 (1.41–3.84)		2.40 (1.45–3.99)		2.56 (1.40–4.67)	
NHR	< 2.58	Reference	0.007^*^	Reference	0.004^*^	Reference	0.014^*^
	≥ 2.58	2.00 (1.21–3.30)		2.14 (1.28–3.57)		2.09 (1.16–3.75)	

Crude model adjusted for no variables. Model 1 adjusted for age and sex. Model 2 adjusted for age, sex, platelet, ALB, FPG, TG, TC, and LDL‐C. ^*^Statistically significant at *p* < 0.05.

Abbreviations: ALB, albumin; FPG, fasting plasma glucose; LDL‐C, low‐density lipoprotein cholesterol; LHR, lymphocyte to high‐density lipoprotein ratio; MHR, monocyte to high‐density lipoprotein ratio; MOH, medication overuse headache; NHR, neutrophil to high‐density lipoprotein ratio; TC, total cholesterol; TG, triglyceride.

## 4 Discussion

This study investigated peripheral indicators of low‐grade inflammation and oxidative stress in MOH and their links to clinical symptoms. Results showed MOH patients had higher MHR, LHR, and NHR values than HC. No significant differences were found in these indicators between the T‐MOH and M‐MOH groups. The TyG index was negatively correlated with headache days per month in MOH patients. Additionally, elevated values of the MHR, LHR, and NHR were independently associated with MOH.

The MHR is an emerging biomarker for inflammation and oxidative stress (Gkantzios et al. 2023). Monocytes play a key role in inflammation by releasing cytokines and mediating immune responses (Guilliams et al. 2018). This study found no significant differences in monocyte counts among MOH, EM, and HC groups, or between T‐MOH and M‐MOH subgroups, consistent with previous research (Forcelini et al. 2011). HDL is known for its protective effects (Tang et al. 2021; Zimetti et al. 2021), but levels were significantly lower in MOH patients compared to HC. We suggested that low HDL may contribute to MOH by causing endothelial dysfunction, similar to its role in increasing migraine risk (Onderwater et al. 2019). MHR is a reliable biomarker for inflammation and oxidative stress. Our study found significantly higher MHR levels in MOH patients and the T‐MOH subgroup compared to HC, with logistic regression showing an independent association between elevated MHR and MOH. This suggested MOH may involve inflammatory and oxidative stress pathways. However, unlike previous research (Ulusoy 2020), we found no significant difference in MHR levels between EM patients and HC, possibly due to differences in sample size and migraine subtypes.

The LHR is emerging as a new inflammation and oxidative stress marker. Lymphocyte counts, crucial for adaptive immunity, showed no significant differences among groups, differing from past findings where MOH patients had higher counts than those with EM (Forcelini et al. 2011). This discrepancy may be attributed to variations in sample size and blood sample timing, such as during interictal periods and headache episodes. Our study found significantly higher LHR levels in MOH patients and the T‐MOH subgroup compared to HC, with high LHR independently linked to MOH. This aligned with previous research on inflammation and oxidative stress in MOH (De Luca et al. 2021; Lucchesi et al. 2015; Vuralli et al. 2024; Wang et al. 2023).

Neutrophils not only promote inflammation by regulating chemokines but also recruit, regulate, and activate the transport of various groups of white blood cells within the tissues (Melo et al. 2019). NHR, the ratio of neutrophil counts to HDL, indicates oxidative stress and inflammation and predicts conditions such as Parkinson's disease, depression, and stroke (Z. Liu et al. 2021; Qing et al. 2024; Wei et al. 2024; Zhang et al. 2022). We found significantly higher NHR values in MOH patients and both the T‐MOH and M‐MOH subgroups compared to the HC group, but no significant difference between T‐MOH and M‐MOH subgroups. Logistic regression indicated that high NHR values were independently associated with MOH. These findings suggested a close association between increased NHR values and MOH, implying that different preexisting headaches like TTH and migraine similarly affected NHR values during MOH development, or that changes in NHR values were more closely related to the pathophysiology of MOH than to the type of preexisting headache.

Unexpectedly, biomarkers like the TyG index, PHR, NAR, NPAR, and PAR showed no significant differences between the MOH, EM, and HC groups, suggesting that inflammation/oxidative stress may not distinctly separate these groups but rather indicate a common low‐grade inflammation in chronic pain (Y. Liu et al. 2023; Sun‐Edelstein et al. 2021). Our results aligned with studies showing no significant link between the TyG index and severe headaches/migraines (Sun et al. 2025; Wu et al. 2025), although they differed from those reporting a significant association (Y. Liu et al. 2023; Yan et al. 2025). Notably, the TyG index showed a negative correlation with monthly headache days in MOH patients, contrary to Wu et al.’s findings of no link with migraine frequency (Wu et al. 2025). These discrepancies could be due to differences in sample size, racial composition, migraine criteria, and chronic pain conditions. This negative correlation suggested that metabolic compensatory mechanisms or confounding factors, like analgesic effects, could influence the relationship, underscoring the complexity of metabolism in MOH (Dağıdır et al. 2023; Grech et al. 2022). Although elevated MHR, NHR, and LHR levels indicated low‐grade inflammation in MOH, their low diagnostic specificity (AUC < 0.6) limited clinical utility. This could be due to MOH's phenotypic variability from different overused medications (Oh et al. 2022), the dominance of central mechanisms (e.g., reward circuit remodeling) over peripheral inflammation (Dai et al. 2021), and confounding lifestyle and emotional factors (Caliri et al. 2021; Ghaemi Kerahrodi and Michal 2020; Shaikh et al. 2024). These biomarkers were less accurate than the ICHD‐III criteria (IHS 2018), which rely on headache history and medication records, in diagnosing MOH. Future research should consider multimodal approaches to integrate biomarker data. Clinically, without reliable biomarkers, detailed medication history and thorough evaluation of clinical symptoms are crucial for accurately identifying MOH patients.

This study has several limitations that warrant cautious interpretation of the results. Being cross‐sectional, it cannot establish a causal relationship between MOH and the examined indicators. Although adjustments were made for age and sex, data on dietary habits, smoking, body mass index, obesity, and emotional states—factors known to affect inflammation and oxidative stress—were not collected (Caliri et al. 2021; Ghaemi Kerahrodi and Michal 2020; Marx et al. 2021; Shaikh et al. 2024). Additionally, the study did not include chronic migraine patients without MOH or conduct a longitudinal follow‐up after detoxification, leaving it unclear whether systemic inflammation and oxidative stress stem from chronic headaches or medication overuse. Finally, the MOH cohort exhibited high compound analgesic use and low triptan use, aligning with previous Chinese studies (Dong et al. 2015; Li et al. 2025). We did not fully analyze the impact of overused acute medications like triptans on inflammatory and oxidative stress markers, despite previous studies reporting their correlations (Puente et al. 2008). However, no significant oxidative stress differences were found between MOH patients overusing triptans and those using NSAIDs, indicating that reduced antioxidant defense in MOH patients was not mainly due to specific medication overuse (Lucchesi et al. 2015). Future research should involve larger, prospective longitudinal studies to confirm these findings.

## 5 Conclusion

In conclusion, MOH patients showed distinct inflammatory and oxidative stress profiles compared to HC, but no significant differences were found between the T‐MOH and M‐MOH subgroups. Elevated LHR, MHR, and NHR were linked to MOH but not to clinical severity. Despite statistical associations with MOH, their low AUC values limited diagnostic utility in clinical practice. Only the TyG index was negatively correlated with monthly headache days in MOH patients. Further research with larger, prospective cohorts is needed to understand the pathophysiological mechanisms of MOH and its connection to inflammation and oxidative stress.

## Author Contributions

**Changling Li**: conceptualization, data curation, formal analysis, investigation, validation, visualization, writing–original draft, writing – review and editing. **Peiqi He**: conceptualization, data curation, formal analysis, investigation, validation, visualization, writing – original draft, writing – review and editing. **Yanbo Li**: conceptualization, investigation, methodology, project administration, supervision, writing – review and editing. **Mengmeng Ma**: conceptualization, funding acquisition, methodology, supervision, writing – review and editing. **Ning Chen**: conceptualization, methodology, supervision, writing – review and editing. **Qian Liu**: formal analysis, methodology, visualization, supervision, writing – review and editing. **Yang Zhang**: formal analysis, methodology, validation, supervision, writing – review and editing. **Xin Jiang**: data curation, investigation, writing – review and editing. **Shiqin Li**: data curation, writing – review and editing. **Hui Lang**: data curation, writing – review and editing. **Jinghuan Fang**: conceptualization, formal analysis, resources, project administration, supervision, writing – review and editing. **Li He**: conceptualization, funding acquisition, resources, project administration, supervision, writing – review and editing.

## Ethics Statement

This cross‐sectional study was approved by the Ethics Committee of West China Hospital, Sichuan University (No. 2020–666).

## Consent

All participants provided verbal informed consent before inclusion.

## Conflicts of Interest

The authors declare no conflicts of interest.

## Peer Review

The peer review history for this article is available at https://publons.com/publon/10.1002/brb3.71004.

## Data Availability

The datasets of the current study could be obtained from the corresponding author upon appropriate request.
